# Perioperative Camrelizumab and Apatinib for High‐Risk Resectable Hepatocellular Carcinoma: A Phase II Study

**DOI:** 10.1002/mco2.70861

**Published:** 2026-10-13

**Authors:** Yunlong Cui, Xu Bao, Ge Yu, Feng Fang, Qiang Li, Han Mu, Chen Liu, Qiang Wu, Wei Zhang, Huikai Li, Ping Chen, Xiaomeng Liu, Hongyuan Zhou, Tianqiang Song

**Affiliations:** ^1^ Department of Hepatobiliary Tumors Tianjin Medical University Cancer Institute and Hospital Tianjin China

**Keywords:** apatinib, camrelizumab, hepatocellular carcinoma, perioperative therapy, pathological response, tumor microenvironment

## Abstract

The optimal perioperative regimen for resectable hepatocellular carcinoma (HCC) remains undefined. This study evaluated the efficacy and safety of perioperative camrelizumab combined with apatinib in patients with resectable high‐risk HCC. In this single‐arm, phase II study, 31 patients with resectable HCC and high recurrence risk factors (e.g., large tumor size, multinodularity, and vascular invasion) received neoadjuvant camrelizumab (200 mg every 2 weeks) and apatinib (250 mg daily) for four cycles, followed by surgical resection and adjuvant therapy with the same regimen for up to 1 year. The primary endpoint was major pathological response (MPR), defined as ≤ 50% viable tumor cells in resected specimens. A total of 31 patients were enrolled in the study. Among 26 patients who underwent surgery, the MPR rate was 46.2%. The median disease‐free survival (DFS) in patients who underwent surgery was 24.1 months (95% confidence interval [CI]: 14.4–not estimable [NE]). The median progression‐free survival (PFS) in all patients was 18.3 months (95% CI: 11.6–NE). In the neoadjuvant setting, Grade 3 or higher adverse events were observed in 12.9% of patients. Perioperative camrelizumab combined with apatinib demonstrated promising efficacy and manageable safety in high‐risk resectable HCC.

## Graphical abstract

In this study, perioperative camrelizumab plus apatinib demonstrated promising efficacy and safety in resectable hepatocellular carcinoma (HCC) patients at high risk of recurrence, achieving a major pathological response of 46.2%. The median disease‐free survival and progression‐free survival were 24.1 months and 18.3 months, respectively. These findings support the potential role of this regimen in improving perioperative management of high‐risk HCC.

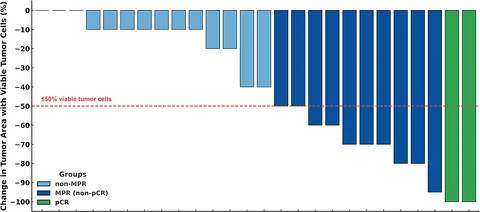

## 1 Introduction

Hepatocellular carcinoma (HCC) accounts for 75%–85% of all primary liver cancers and remains the third leading cause of cancer‐related mortality worldwide [1]. It is particularly prevalent in Asia, where approximately 72% of cases are reported, with chronic hepatitis B virus (HBV) infection being the primary risk factor [1, 2]. Despite advances in early diagnosis and treatment, only about 30% of patients with HCC are diagnosed at a stage amenable to surgical resection [3]. In patients with preserved liver function and resectable tumors, surgery offers a relatively favorable prognosis, with 5‐year survival rates reaching approximately 70% [4]. However, the high rate of tumor recurrence, estimated at 70% within 5 years post‐resection [5], poses a significant challenge to long‐term survival and patient quality of life. Recurrence after resection is frequently driven by factors such as portal vein tumor thrombus (PVTT), microvascular invasion (MVI), and the presence of satellite nodules, which are considered high‐risk indicators for postoperative relapse [6]. While negative surgical margins are typically achieved during resection, it is widely believed that recurrence arises from undetected micrometastases that persist beyond the resection margins [7]. As such, perioperative therapeutic strategies that target micrometastatic disease hold considerable promise in improving outcomes for patients with resectable HCC.

In recent years, the combination of immunotherapy and anti‐angiogenic therapy has garnered significant interest as a promising treatment strategy for HCC [8]. The IMbrave150 trial demonstrated the benefit of atezolizumab in combination with bevacizumab as a first‐line treatment for patients with unresectable HCC, showing improvements in overall survival (OS) compared to sorafenib [9, 10]. The IMbrave050 study recently showed that for patients at high risk of recurrence following curative‐intent resection or ablation, the combination of atezolizumab and bevacizumab significantly improved recurrence‐free survival (RFS) compared to active surveillance [11]. Despite these promising results in advanced and high‐risk HCC, the use of neoadjuvant or perioperative immunotherapy for resectable HCC remains an area of limited exploration. While a few early‐stage studies have reported some evidence of benefit, these studies have been constrained by small sample sizes and preliminary findings [12, 13, 14, 15].

Camrelizumab, a PD‐1 inhibitor, and apatinib, a tyrosine kinase inhibitor (TKI) targeting vascular endothelial growth factor (VEGF) receptor 2, represent two promising therapeutic agents in the treatment of HCC [16, 17]. Recent results from the phase 3 CARES‐310 trial demonstrated that the combination of camrelizumab and apatinib significantly improved progression‐free survival (PFS) and OS compared to sorafenib in patients with unresectable HCC, establishing it as an effective first‐line treatment for this patient population [18]. Furthermore, the phase 2/3 CARES‐009 trial (NCT04521153) recently demonstrated that perioperative camrelizumab and apatinib significantly improved clinical outcomes in patients with resectable HCC at intermediate or high risk of recurrence [19]. However, the optimal neoadjuvant treatment strategy, including the number of treatment cycles, remains to be determined. This study aims to evaluate the efficacy and safety of perioperative camrelizumab in combination with apatinib in patients with resectable HCC and high recurrence risk.

## 2 Results

### 2.1 Patient Characteristics

Between March 2021 and August 2022, 31 patients were enrolled in the study. All patients received neoadjuvant therapy and were included in both the full analysis set (FAS) and the safety analysis set (SS). Of these, 26 patients underwent surgical resection and were included in the efficacy analysis set (EAS), while 22 patients proceeded to receive adjuvant therapy (Figure 1). Among the five patients who did not undergo surgery, three experienced radiological disease progression and two declined surgery due to adverse events (AEs). Specifically, three patients experienced radiological intrahepatic disease progression during neoadjuvant treatment (two after completing four cycles and one after three cycles of camrelizumab plus apatinib). Following progression, two patients received transarterial chemoembolization (TACE), with one subsequently switching to lenvatinib, while the third patient was treated with a combination of TACE, lenvatinib, and a PD‐1 inhibitor. The remaining two patients discontinued neoadjuvant therapy early due to AEs. One patient developed immune‐mediated myocarditis after one cycle and was lost to follow‐up after hospital discharge without available radiological response assessment. The other patient developed myasthenia gravis after one cycle of therapy and, due to COVID‐19 lockdown restrictions, continued oral apatinib alone; this patient was also lost to follow‐up without evaluable tumor assessment.

**FIGURE 1:**
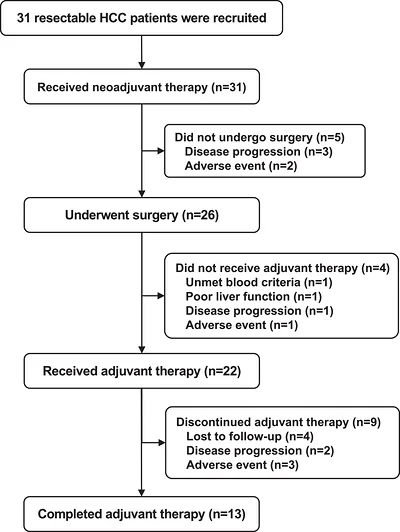
Patient flowchart. HCC: hepatocellular carcinoma.

The median age was 54 years (IQR 52–62), and the majority were male (77.4%). According to the Chinese Liver Cancer (CNLC) staging system, nearly half of the patients were classified as stage Ib (48.4%), with the remainder distributed across stages IIa (12.9%), IIb (16.1%), and IIIa (22.6%). Similarly, according to the Barcelona Clinic Liver Cancer (BCLC) staging system, 51.6% of patients were classified as stage A, followed by stage B (25.8%) and stage C (22.6%). All patients had preserved liver function with Child‐Pugh class A scores. HBV infection was present in 71% of the cohort, and 54.8% had underlying liver cirrhosis. Ascites was absent in the majority of patients (87.1%), and PVTT was identified in 22.6% of cases (Table 1).

**TABLE 1:** Baseline characteristics of patients.

Variables	All (*n* = 31)
Age, years, median (interquartile range)	54 (52,62)
Sex, *n* (%)	
Male	24 (77.4%)
Female	7 (22.6%)
ECOG PS	
0	18 (58.1%)
1	13 (41.9%)
CNLC stage, *n* (%)	
Ib	15 (48.4%)
IIa	4 (12.9%)
IIb	5 (16.1%)
IIIa	7 (22.6%)
BCLC stage, *n* (%)	
A	16 (51.6%)
B	8 (25.8%)
C	7 (22.6%)
Number of tumors	
1	21 (67.7%)
2	5 (16.1%)
3	2 (6.5%)
4	3 (9.7%)
Child‐Pugh class A, *n* (%)	31 (100%)
Baseline AFP >400 ng/mL, *n* (%)	12 (38.7%)
HBV infection, *n* (%)	22 (71.0%)
Cirrhosis, *n* (%)	17 (54.8%)
Ascites, *n* (%)	4 (12.9%)
PVTT, *n* (%)	7 (22.6%)

Abbreviations: AFP: alpha‐fetoprotein; BCLC: Barcelona Clinic Liver Cancer stage; CNLC: Chinese Liver Cancer stage; ECOG PS: Eastern Cooperative Oncology Group performance status; HBV: hepatitis B virus; PVTT: portal vein tumor thrombus.

### 2.2 Efficacy

Among the 26 patients who underwent surgery, complete response rate (pCR) was achieved in two patients (7.7%), while major pathological response (MPR) was observed in 12 patients (46.2%) (Table 2, Figure S1). The MPR rate was 46.2% (95% confidence interval [CI], 27.0%–65.0%) in EAS and 38.7% (95% CI, 21.5%–55.9%) in FAS. In the FAS, based on Response Evaluation Criteria in Solid Tumors (RECIST) version 1.1, partial response was achieved in four patients (12.9%), stable disease in 21 patients (67.7%), and progressive disease in four patients (12.9%). The objective response rate (ORR) was 12.9%, and the disease control rate (DCR) was 80.6%. Using modified RECIST (mRECIST) criteria, the ORR and DCR were 45.2% and 83.9%, respectively (Table S1). The best percentage changes in baseline target lesion size are shown in Figure S2.

**TABLE 2:** Pathological response in the efficacy analysis set.

Outcomes, *n* (%)	All (*n* = 26)
Pathological response, *n* (%, 95%CI)	
pCR	2 (7.7%, 1.0%–25.1%)
MPR	12 (46.2%, 27.0%–65.0%)
MV1 grading, *n* (%)	
M0	15 (57.7%)
M1	9 (34.7%)
M2	2 (7.7%)

Abbreviations: CI: confidence interval; MPR: major pathological response; pCR: pathological complete response.

As of the cut‐off date of December 11, 2024, with a median follow‐up of 39.3 months, 26 patients had experienced 14 disease‐free survival (DFS) events. The median DFS was 24.1 months (95% CI: 14.4–not estimable [NE]). The 1‐year DFS rate was 69.2% (95% CI: 53.6%–89.5%), and the 3‐year DFS rate was 46.2% (95% CI: 30.5%–70.0%). In all patients, 17 PFS events were observed, with a median PFS of 18.3 months (95% CI: 11.6–NE). The 1‐year PFS rate was 62.5% (95% CI: 47.2%–82.8%), and the 3‐year PFS rate was 41.7% (95% CI: 27.1%–64.1%) (Figure 2).

**FIGURE 2:**
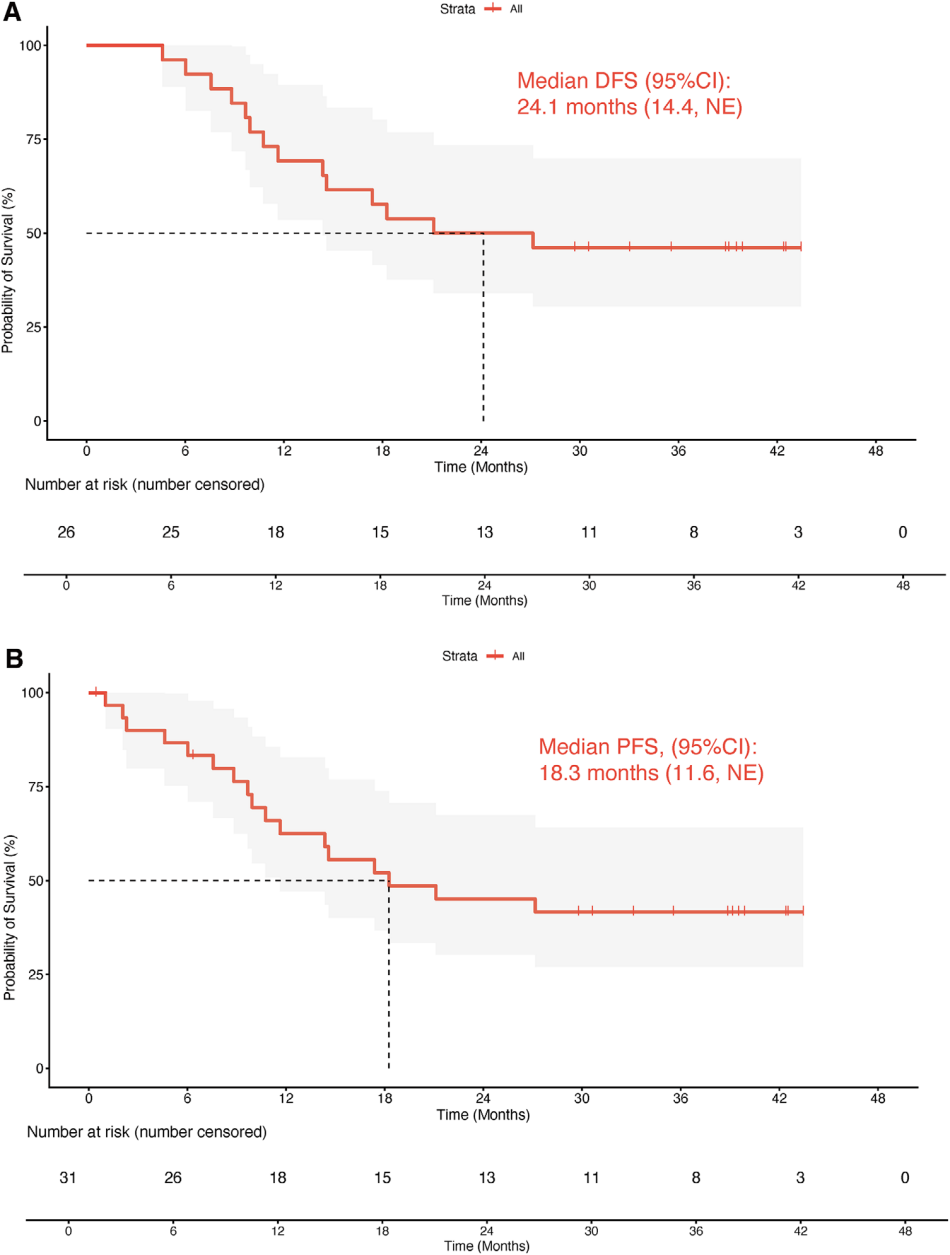
Kaplan–Meier curves for (A) disease‐free survival (DFS) in the efficacy analysis set (single‐group stratum, *n* = 26) and (B) progression‐free survival (PFS) in all enrolled patients (single‐group stratum, *n* = 31). CI, confidence interval; NE, not estimable.

When stratified by pathological response, patients achieving MPR demonstrated a numerically longer DFS compared with non‐MPR patients (median DFS: not reached [95% CI, 18.3–NE] vs. 15.9 months [95% CI, 9.7–NE], respectively). However, this difference did not reach statistical significance (log‐rank *p* = 0.1576; non‐MPR vs. MPR: hazard ratio = 2.16, 95% CI: 0.72–6.47) (Figure 3).

**FIGURE 3:**
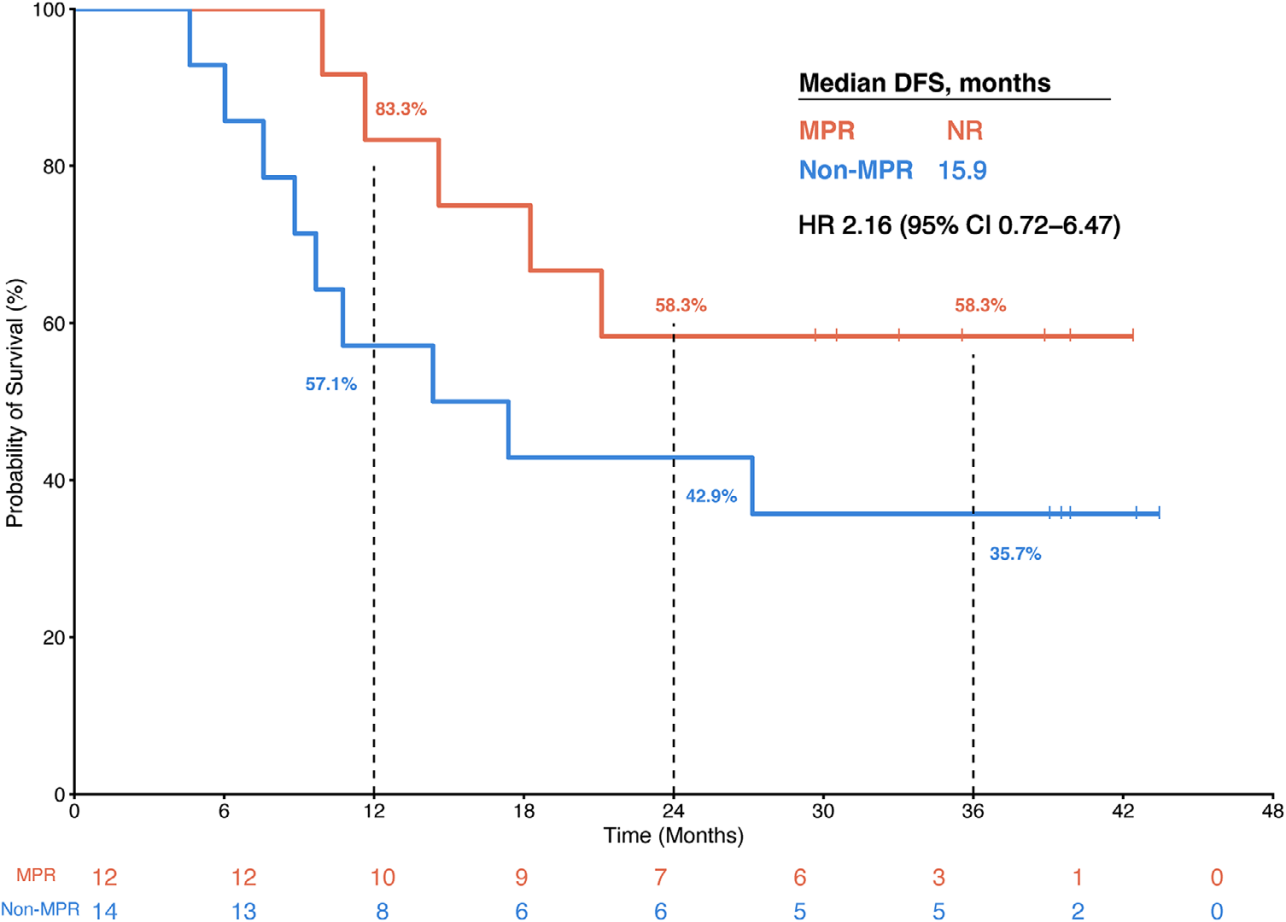
Kaplan–Meier curves for disease‐free survival in patients with or without major pathological response (MPR) in the efficacy analysis set. CI, confidence interval; HR, hazard ratio; NR, not reached.

### 2.3 Safety

In the neoadjuvant setting (*n* = 31), 29 patients (93.5%) experienced AEs of any grade, with four patients (12.9%) experiencing Grade 3 or higher AEs. The most frequent grade 3 or higher AEs included aspartate aminotransferase (AST) increased (9.7%), alanine aminotransferase (ALT) increased (9.7%), and conjugated bilirubin increased (3.2%) (Table 3). After surgery (*n* = 26), all patients experienced AEs, with 22 patients (84.6%) reporting grade 3 or higher AEs. The most frequent grade 3 or higher AEs included AST increased (76.9%), ALT increased (46.2%), and conjugated bilirubin increased (23.1%) (Table S2). One patient discontinued neoadjuvant therapy due to a rash, and three patients discontinued adjuvant therapy due to AEs. Importantly, no treatment‐related deaths occurred during the study period. Temporary treatment delays due to AEs occurred in two patients during the neoadjuvant phase. In the adjuvant phase, 16 patients experienced delays in apatinib and 17 in camrelizumab; these events were manageable with supportive care, and treatment was resumed after recovery.

**TABLE 3:** Preoperative adverse events occurring in at least 10% of patients (*n* = 31).

Events, *n* (%)	Any grade	Grade 3–4
Any events	29 (93.5%)	4 (12.9%)
Hypoalbuminemia	18 (58.1%)	0
Conjugated bilirubin increased	14 (45.2%)	1 (3.2%)
Alanine aminotransferase increased	12 (38.7%)	3 (9.7%)
Platelet count decreased	12 (38.7%)	0
Lymphocyte count decreased	11 (35.5%)	1 (3.2%)
Total bilirubin increased	11 (35.5%)	0
Aspartate aminotransferase increased	11 (35.5%)	3 (9.7%)
Anemia	10 (32.3%)	1 (3.2%)
Neutrophil count decreased	9 (29.0%)	0
Leukocyte count decreased	9 (29.0%)	0
γ‐Glutamyl transferase increased	8 (25.8%)	0
Lactate dehydrogenase increased	7 (22.6%)	0
Alkaline phosphatase increased	5 (16.1%)	0
Eosinophil count increased	5 (16.1%)	0
Creatine kinase increased	5 (16.1%)	0
Hypertriglyceridemia	4 (12.9%)	0
Hyponatremia	4 (12.9%)	0
Hypocalcemia	4 (12.9%)	0

## 3 Discussion

This study evaluated the efficacy and safety of perioperative camrelizumab combined with apatinib in patients with resectable HCC at high risk of recurrence. Our findings demonstrate that this combination therapy yields favorable clinical outcomes, with 46.2% of patients achieving MPR. These results highlight the potential of perioperative immunotherapy in improving outcomes in high‐risk resectable HCC.

Although systemic therapies are not yet standard in the neoadjuvant setting for HCC, emerging trials suggest significant benefits for patients undergoing resection. In a phase II trial, 27 patients with resectable HCC received neoadjuvant and adjuvant nivolumab with or without ipilimumab. Among the 20 patients who underwent resection, six achieved MPR and five had pCR. Additionally, the combination of nivolumab and ipilimumab showed improved PFS compared to nivolumab alone [13]. A phase II study of perioperative camrelizumab and apatinib in 18 patients with resectable HCC showed that four patients had more than 90% tumor necrosis, and one achieved pCR. Among the 13 patients who received adjuvant therapy, the 1‐year RFS rate was 53.9%, with fewer recurrences in patients with higher degrees of tumor necrosis [12]. Similarly, a trial of cemiplimab, another PD‐1 inhibitor, in 21 patients with resectable HCC showed that four patients achieved > 70% tumor necrosis and seven had > 50% necrosis [15]. In our study, the combination of camrelizumab and apatinib demonstrated encouraging clinical efficacy, with 46.2% of patients achieving MPR and a median DFS of 24.1 months. This combination therapy enhanced the immune cell infiltration into the tumor microenvironment, facilitated by anti‐angiogenic effects that reduce vascular density and promote tumor vessel normalization. This, in turn, improves the delivery and efficacy of immunotherapy, enabling a more robust anti‐tumor immune response [20, 21]. Additionally, the reduction in tumor‐associated immunosuppression, particularly through the modulation of tumor‐associated macrophages and regulatory T cells, may further amplify the effectiveness of immunotherapy, leading to improved clinical outcomes [22]. More recently, the phase 2/3 CARES‐009 trial further validated the clinical benefit of perioperative camrelizumab and apatinib in resectable HCC, demonstrating improved outcomes compared with standard approaches [19]. These findings are consistent with our results, supporting the role of perioperative strategies in this population. Notably, CARES‐009 employed a shorter neoadjuvant regimen of two cycles, whereas our study used four cycles of camrelizumab combined with apatinib. The optimal duration and intensity of neoadjuvant therapy remain uncertain, and whether extended treatment may enhance pathological response without compromising surgical feasibility warrants further investigation in future studies.

Pathological response has gained increasing recognition as a potential surrogate endpoint for survival in cancer immunotherapy, surpassing radiological assessment in prognostic value, particularly in cancers such as melanoma and lung cancer [23, 24]. In HCC, the clinical significance of pathological response remains incompletely defined, particularly in the neoadjuvant setting. In this study, MPR was defined as ≤ 50% residual viable tumor cells, consistent with CNLC guideline recommendations [25] and prior studies in resectable HCC [19]. In our study, using the 50% cutoff, patients achieving MPR demonstrated a numerically longer DFS compared with non‐MPR patients (median DFS not reached vs. 15.9 months), although this difference did not reach statistical significance, which may be due to the limited sample size and event number in the present study. Evaluation of deeper pathological responses was constrained by the small number of patients achieving ≥ 90% tumor necrosis (*n* = 3), precluding meaningful statistical analysis. These findings highlight the ongoing uncertainty regarding the optimal pathological response cutoff in HCC. Future prospective studies with sufficient sample size are required to establish standardized, outcome‐validated pathological response criteria for neoadjuvant therapy in HCC.

The definition of high‐risk recurrence factors is critical in designing perioperative studies for HCC, particularly in populations with more inclusive resection criteria, as seen in China. Compared with European and US guidelines, which recommend stricter criteria for surgical resection, Asian guidelines often allow for resection in cases with larger or multiple tumors [7]. This broader eligibility inevitably results in a higher proportion of patients with advanced disease characteristics, contributing to lower reported 5‐year survival rates of approximately 50% in China, compared with 70% in Europe [7]. Identifying patients at high risk of recurrence is essential to optimize the benefits of perioperative therapies. In our study, high‐risk recurrence factors were defined as stage Ib (single tumor > 6.5 cm or two to three tumors ≤ 3 cm), stage IIa (two to three tumors > 3 cm), stage IIb (≥ 4 tumors), or stage IIIa (visible vascular invasion). Consistent with prior studies, tumor size, number of lesions, elevated alpha‐fetoprotein (AFP) levels, and vascular invasion were strongly associated with early recurrence [26]. Additionally, clinical parameters such as male gender, high albumin‐bilirubin (ALBI) grade, and multifocal disease have been implicated as predictors of early relapse [27]. The inclusion of high‐risk patients in perioperative studies is crucial, as these individuals are more likely to derive substantial benefits from adjuvant or neoadjuvant therapies. However, further research is needed to refine risk stratification models and validate the utility of these criteria in predicting outcomes.

The safety profile observed in this study was consistent with previous reports of camrelizumab combined with apatinib in HCC [12, 18], with no new safety concerns identified. AEs were manageable and aligned with the known toxicities of this combination therapy. Grade 3 or higher AEs were also within expectations, with the most frequent events being elevated AST and elevated ALT. Importantly, there were no treatment‐related deaths. Notably, neoadjuvant therapy with camrelizumab and apatinib did not compromise the feasibility of surgery, as the majority of patients successfully underwent resection without delays or significant intraoperative complications. Furthermore, the manageable safety profile underscores the tolerability of this regimen and supports its use in the perioperative setting for high‐risk HCC patients.

This study has several limitations that should be acknowledged. This was a single‐center trial with a small sample size. While our findings suggest a promising role for camrelizumab combined with apatinib in the perioperative management of high‐risk HCC, randomized controlled trials are essential to confirm the efficacy and safety of this approach and establish its role in clinical practice.

## 4 Conclusion

In this study, perioperative camrelizumab combined with apatinib demonstrated promising efficacy and safety in resectable HCC patients at high risk of recurrence, achieving an MPR of 46.2%. While further randomized studies are needed, these findings support the potential role of this regimen in improving perioperative management of high‐risk HCC.

## 5 Materials and Methods

### 5.1 Study Design and Patients

This was a single‐arm, phase 2 study. Patients aged 18–75 years with histologically confirmed HCC and lesions meeting the surgical resection criteria specified in the 2019 Standardization for Diagnosis and Treatment of Hepatocellular Carcinoma guidelines [28] were included. Eligible patients had an Eastern Cooperative Oncology Group performance status of 0–1, Child‐Pugh class A liver function, and a predicted survival of at least 6 months. Preoperative risk assessment identified at least one of the following high‐risk recurrence factors: stage Ib (single tumor > 6.5 cm or two to three tumors ≤ 3 cm); stage IIa (two to three tumors > 3 cm); stage IIb (≥ 4 tumors); or stage IIIa (visible vascular invasion). Additionally, patients were required to have at least one measurable lesion based on RECIST version 1.1, with no prior local therapies to the lesion. Key exclusion criteria included prior systemic therapies, concurrent active malignancies, and significant comorbid conditions such as uncontrolled hypertension, active gastrointestinal bleeding, or thromboembolic events within 12 months.

The trial was conducted in accordance with the Declaration of Helsinki and approved by the ethics committee of Tianjin Medical University Cancer Institute and Hospital. Written informed consent was obtained from all participants. The trial was registered on ClinicalTrials.gov (NCT04701060).

### 5.2 Procedures

Eligible patients received four cycles of neoadjuvant therapy comprising oral apatinib (250 mg once daily) and intravenous camrelizumab (200 mg administered on Day 1 of a 14‐day cycle). Following completion of neoadjuvant therapy, patients underwent hepatectomy. Adjuvant therapy commenced 4–8 weeks after surgery, consisting of apatinib (250 mg once daily) and camrelizumab (200 mg every 2 weeks), continuing for up to 1 year, until disease progression, or the development of unacceptable toxicity.

Surgical procedures were performed according to standard hepatobiliary oncologic practice. All operable patients underwent curative‐intent (R0) resection after completion of neoadjuvant therapy. The extent of resection was determined based on tumor characteristics and liver functional reserve, as assessed by preoperative imaging performed after neoadjuvant treatment, together with intraoperative findings. When anatomically feasible, a target surgical margin of at least 1 cm was pursued. Intraoperative ultrasound was routinely used to guide resection and to confirm tumor boundaries. For patients who achieved radiological complete response according to mRECIST criteria (no patient achieved complete response according to RECIST version 1.1 in this study), surgical resection was also performed based on the tumor location and extent identified on post‐neoadjuvant preoperative imaging. Pathological margin status was confirmed postoperatively. Pathological evaluation was performed on surgically resected specimens. All sections corresponding to pre‐treatment imaging lesions were reviewed by hepatobiliary pathologists to determine the proportion of viable tumor cells. The degree of necrosis was calculated as the ratio of viable tumor cells to the total tumor area.

Tumor responses were assessed using RECIST version 1.1 criteria. Both contrast‐enhanced computed tomography and magnetic resonance imaging were permitted. Baseline imaging was performed within 4 weeks prior to the initiation of treatment. Tumor response was assessed at the end of the neoadjuvant phase (after four cycles) to guide surgical decision‐making. During adjuvant therapy, imaging assessments were conducted every 6 weeks for the first three evaluations and every 9 weeks thereafter. In cases of suspected disease progression according to RECIST version 1.1, confirmation was required with mRECIST criteria using clinical imaging performed 4 weeks later. AEs were recorded and graded according to the National Cancer Institute Common Terminology Criteria for Adverse Events, version 5.0.

### 5.3 Endpoints

The primary endpoint was MPR, defined as ≤ 50% viable tumor cells in the resected specimen, in accordance with the CNLC guidelines and prior neoadjuvant HCC studies [25, 29]. Secondary endpoints included the pCR, defined as the absence of residual viable tumor cells in the resected specimen. Additional secondary endpoints included the ORR following neoadjuvant therapy and prior to surgery, calculated as the sum of complete response and partial response rates based on RECIST version 1.1 and mRECIST. The DCR was defined as the proportion of patients achieving complete response, partial response, or stable disease. DFS was defined as the time from surgery to the first documented recurrence, distant metastasis, or death from any cause. PFS was defined as the time from the initiation of neoadjuvant therapy to radiological disease progression or death. Safety was also a secondary endpoint.

### 5.4 Statistical Analysis

Sample size calculation was based on an expected improvement in MPR from 25% [30] to 50%. Using a two‐sided alpha level of 0.05 and a power of 80%, the required sample size was calculated to be 26 patients. To account for a 15% dropout rate, 30 patients were enrolled. The FAS included all patients who received at least one dose of the study drug, while the EAS comprised patients who completed HCC resection. The SS included all participants who received at least one dose of the study drug. Continuous variables were presented as medians with interquartile ranges (IQRs), while categorical variables were expressed as counts and percentages. The 95% CIs for MPR, pCR, ORR, and DCR were estimated using the Clopper–Pearson method. Survival outcomes, including DFS and PFS, were analyzed using the Kaplan–Meier method, with median values and 95% CIs reported. DFS was assessed in EAS, whereas PFS was assessed in FAS. A post hoc analysis of DFS was performed comparing patients with MPR and non‐MPR. Group differences were assessed using the log‐rank test. Hazard ratios and corresponding 95% CIs were estimated using a Cox proportional hazards model. All statistical tests were two‐sided, and a *p* value < 0.05 was considered statistically significant. All statistical analyses were performed using SAS software.

## Author Contributions

Conceptualization: Yunlong Cui and Tianqiang Song. Methodology: Yunlong Cui and Tianqiang Song. Formal analysis: Xu Bao. Investigation: Yunlong Cui, Xu Bao, Ge Yu, Feng Fang, Qiang Li, Han Mu, Chen Liu, Qiang Wu, Wei Zhang, Huikai Li, Ping Chen, Xiaomeng Liu, Hongyuan Zhou, and Tianqiang Song. Data curation: Xu Bao. Resources: Xu Bao. Investigation: Yunlong Cui, Xu Bao, Ge Yu, Feng Fang, Qiang Li, Han Mu, Chen Liu, Qiang Wu, Wei Zhang, Huikai Li, Ping Chen, Xiaomeng Liu, Hongyuan Zhou, and Tianqiang Song. Project administration: Tianqiang Song. Supervision: Tianqiang Song. Writing – original draft: Yunlong Cui. Writing – review and editing: Xu Bao. Investigation: Yunlong Cui, Xu Bao, Ge Yu, Feng Fang, Qiang Li, Han Mu, Chen Liu, Qiang Wu, Wei Zhang, Huikai Li, Ping Chen, Xiaomeng Liu, Hongyuan Zhou, and Tianqiang Song. All authors have read and approved the final manuscript.

## Funding

This study was funded by Jiangsu Hengrui Pharmaceuticals Co., Ltd., which provided the study drugs.

## Ethics Statement

The trial was approved by the ethics committee of Tianjin Medical University Cancer Institute and Hospital, Tianjin, China on February 4, 2021 (protocol number: E2020699A). Written informed consent was obtained from all participants.

## Conflicts of Interest

The authors declare no conflicts of interest.

## Supporting information




**Supporting File 1**: mco270861‐sup‐0001‐SuppMat.docx

## Data Availability

Deidentified participant data from this study will be made available to qualified researchers upon reasonable request. Requests should be directed to the corresponding author via email.
