# Supplementary material for: Perioperative Camrelizumab and Apatinib for High‐Risk Resectable Hepatocellular Carcinoma: A Phase II Study
Source: MedComm (2020). 2026 Oct 13;7(10):e70861. doi: 10.1002/mco2.70861 (PMC13660721; doi:10.1002/mco2.70861)
Supplement: Supplementary file 1 [file MCO2-7-e70861-s001.docx]

**Perioperative Camrelizumab and Apatinib for High-Risk Resectable Hepatocellular Carcinoma: A Phase II Study**

**Running title:** **Camrelizumab plus Apatinib for HCC**

Yunlong Cui 1#; Xu Bao 1#; Ge Yu 1; Feng Fang 1; Qiang Li 1; Han Mu 1; Chen Liu 1; Qiang Wu 1; Wei Zhang 1; Huikai Li 1; Ping Chen 1; Xiaomeng Liu 1; Hongyuan Zhou 1; Tianqiang Song 1*

1. Department of Hepatobiliary Tumors, Tianjin Medical University Cancer Institute and Hospital, Tianjin, China

#These authors contributed equally to this work.

**Corresponding Author:**

Tianqiang Song, MD

Department of Hepatobiliary Tumors, Tianjin Medical University Cancer Institute and Hospital,

Huanhuxi Road, Hexi District, Tianjin, China, 300060

Phone: +86-186-2222-1077

Email: tjchi@hotmail.com

**Table S1.** Tumor response in the full analysis set

| Response, n (%) | RECIST v1.1 (n=31) | mRECIST (n=31) |
| --- | --- | --- |
| Best of response |  |  |
| Complete response | 0 | 2 (6.5%) |
| Partial response | 4 (12.9%) | 12 (38.7%) |
| Stable disease | 21 (67.7%) | 12 (38.7%) |
| Progressive disease | 4 (12.9%) | 3 (9.7%) |
| Not evaluable | 2 (6.5%) | 2 (6.5%) |
| Objective response rate | 4 (12.9%) | 14 (45.2%) |
| 95%CI | 3.6%-29.8% | 27.3%-64.0% |
| Disease control rate | 25 (80.6%) | 26 (83.9%) |
| 95%CI | 62.5%-92.6% | 66.3%-94.6% |

RECIST v1.1: response evaluation criteria in solid tumors version 1.1; mRECIST: modified response evaluation criteria in solid tumors; CI: confidence interval.

**Figure S1:** Depth of pathological regression in primary tumor. MPR: major pathological response; pCR: pathological complete response.

**Figure S2:** Best percentage changes from baseline target lesion size according to (A) RECIST v1.1 and (B) mRECIST. CR: complete response; PR: partial response; SD: stable disease; PD: progressive disease.

**Table S2.** Postoperative adverse events occurring in at least 10% of patients (n=26).

| Events, n (%) | Any grade | Grade 3-4 |
| --- | --- | --- |
| Any events | 26 (100.0%) | 22 (84.6%) |
| Alanine aminotransferase increased | 26 (100.0%) | 12 (46.2%) |
| Aspartate aminotransferase increased | 26 (100.0%) | 20 (76.9%) |
| Hypoalbuminemia | 25 (96.2%) | 0 |
| Conjugated bilirubin increased | 24 (92.3%) | 6 (23.1%) |
| Anemia | 23 (88.5%) | 1 (3.8%) |
| Hypocalcemia | 21 (80.8%) | 0 |
| Lymphocyte count decreased | 20 (76.9%) | 5 (19.2%) |
| Platelet count decreased | 20 (76.9%) | 2 (7.7%) |
| Hypophosphatemia | 19 (73.1%) | 0 |
| Total bilirubin increased | 17 (65.4%) | 2 (7.7%) |
| Creatine kinase increased | 15 (57.7%) | 2 (7.7%) |
| Hyponatremia | 14 (53.8%) | 0 |
| γ-glutamyl transferase increased | 14 (53.8%) | 3 (11.5%) |
| Hyperglycemia | 13 (50.0%) | 0 |
| Lactate dehydrogenase increased | 13 (50.0%) | 0 |
| Alkaline phosphatase increased | 10 (38.5%) | 0 |
| Neutrophil count decreased | 8 (30.8%) | 1 (3.8%) |
| Fibrinogen decreased | 8 (30.8%) | 0 |
| International normalized ratio increased | 8 (30.8%) | 0 |
| Hypochloremia | 7 (26.9%) | 0 |
| Hyperuricemia | 5 (19.2%) | 0 |
| Hypomagnesemia | 5 (19.2%) | 0 |
| Hypokalemia | 5 (19.2%) | 1 (3.8%) |
| Leukocyte count decreased | 4 (15.4%) | 1 (3.8%) |
| Activated partial thromboplastin time prolonged | 4 (15.4%) | 0 |
| Serum amylase increased | 4 (15.4%) | 0 |
| Hemoglobin increased | 3 (11.5%) | 0 |
| Eosinophil count increased | 3 (11.5%) | 0 |
| Creatinine increased | 3 (11.5%) | 0 |
| Proteinuria | 3 (11.5%) | 0 |
| Hypertriglyceridemia | 3 (11.5%) | 1 (3.8%) |
